# Moldflow Simulation and Characterization of Pure Copper Fabricated via Metal Injection Molding

**DOI:** 10.3390/ma16155252

**Published:** 2023-07-26

**Authors:** Warda Bahanan, Siti Fatimah, Hyunseok Song, Eun Hye Lee, Dong-Ju Kim, Hae Woong Yang, Chang Hoon Woo, Jungho Ryu, I Putu Widiantara, Young Gun Ko

**Affiliations:** 1 School of Materials Science and Engineering, Yeungnam University, Gyeongsan 38541, Republic of Korea; 2 Kyerim Metal Co., Ltd., Chilgok 39910, Republic of Korea; 3 SeA Mechanics Co., Ltd., Gumi 39379, Republic of Korea; 4 Pohang Institute of Metal Industry Advancement, Pohang 37666, Republic of Korea

**Keywords:** copper, metal injection molding, moldflow, green part

## Abstract

Metal injection molding (MIM) is a representative near-net-shape manufacturing process that fabricates advanced geometrical components for automobile and device industries. As the mechanical performance of an MIM product is affected by green-part characteristics, this work investigated the green part of pure copper processed with MIM using the injection temperature of ~180 °C and injection pressure of ~5 MPa. A computational analysis based on the Moldflow program was proposed to simulate the effectivity of the process by evaluating the confidence of fill, quality prediction, and pressure drop of three distinctive regions in the green part. The results showed that the ring and edge regions of the green parts showed localized behavior, which was related to processing parameters including the position of the gate. A microstructural observation using scanning electron microscopy and a 3D X-ray revealed that both the surface and body matrix consisted of pores with some agglomeration of micro-pores on the edges and ring part, while any critical defects, such as a crack, were not found. A microhardness analysis showed that the three regions exhibited a reasonable uniformity with a slight difference in one specific part mainly due to the localized pore agglomeration. The simulation results showed a good agreement with the microstructures and microhardness data. Thus, the present results are useful for providing guidelines for the sound condition of MIM-treated pure copper with a complex shape.

## 1. Introduction

Pure copper is one of the most widely employed non-ferrous metals in modern industries due to its excellent thermal and electrical characteristics [1,2,3,4]. In addition, copper exhibits a high specific strength, good corrosion resistance, and is classified as commercially low-cost, enabling its pure form to exhibit versatility across various applications [5,6]. However, current rapid industrial development requires copper components to have sophisticated shapes, so cutting-edge manufacturing techniques must be used [1,2,3,4]. Metal injection molding (MIM) is an advanced-shape manufacturing process that merges plastic injection molding and powder metallurgy. MIM offers the ability to produce small parts with a unique set of desirable uncommon geometry; characteristics, including cost effectiveness through the use of inexpensive materials; efficient production methods; a wide range of composition choices; and a high density with favorable mechanical properties in comparison to wrought materials [7,8]. The MIM route consists of four distinct stages: (i) the mixing of metallic powder and a binder to produce a feedstock; (ii) the injection of the feedstock into a mold that is giving shape to a green part; (iii) the removal of the binder (debinding), which later forms a brown part; and (iv) sintering for reducing the porosity [9]. Each step leads to a greater surface finish and highly dense structure [10]. In the case of MIM using two-tube channels, the feedstock is usually injected from the center towards two different ends during the molding process in stage 2. The feedstock flowing from the middle toward the end of the two-tube channels is considered as the best scenario for reducing the flow length, minimizing the gradient in terms of pressure, temperature, etc. [11]. Consequently, a pressure drop, which is known to have potential to develop defects such as short shots, warpage, density differences, air traps, etc. [12,13,14], is reduced accordingly, albeit not eliminated completely. MIM parameters, namely the injection pressure, injection temperature, and injection gate location [15], can be controlled and their optimization [16] can effectively alleviate the problem encountered during MIM. Critical process parameters in MIM play a significant role in achieving a defect-free final product [15,17,18,19], including factors like the temperature, pressure, filling rate, and shear deformation rate [20,21]. These parameters are crucial for obtaining a high-quality end product with minimal to no flaws.

Up until now, various industrial designs based on actual experiments at several high temperatures, pressures, etc., have been made, which rendered the cost of time and effort of equipment in actual industrial fields. In spite of industrial importance, no study has been reported on pure copper with the aid of computational prediction and merely relied on case-by-case works [22,23]. According to a review of metal processing employing MIM on an industrial scale, simulations ought to be implemented for quality control considerations in order to convince society to adopt the MIM approach [15].

Therefore, the present study scrutinizes the role of MIM parameters, which comprise the injection pressure, injection temperature, and gate location, in the second stage that produces the copper green-part object. The MIM parameters for the second stage are considered very crucial since they affect the quality of the product after debinding and sintering [17], and will be discussed further in the present work. Here, the designed product (case code name: outlet-combo charger terminal body) was made of pure copper, which consisted of two-tube channels with different wall thicknesses and diameters joined together in the middle with ring-shaped reinforcement (the details are shown in the following section). We simulated the MIM process using Moldflow, considering its superiority in predicting the flow pattern and providing a quick result [24,25].

Scanning electron microscopy and 3D X-ray computed tomography were used to characterize the surface properties and three-dimensional projection of the interior of the MIM final product. From this study, we hope to establish the guidelines for processing copper via the MIM method.

## 2. Materials and Methods

### 2.1. Feedstock

**Figure 1:**
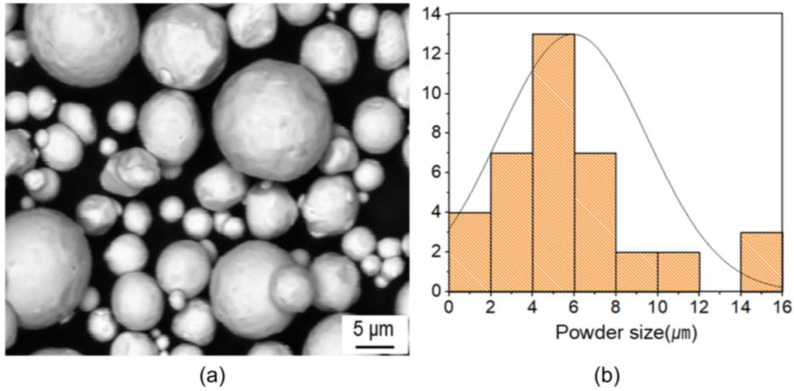
(**a**) SEM image of spherical precursor powder of copper feedstock and (**b**) powder size distribution.

Pure copper powder manufactured by Changseong Co., Ltd. (Incheon, Republic of Korea) with an average size and density of ~5.2 µm (the size variance was from 1 µm to 15 µm) and 4.9 g/cm^3^, respectively, was mixed mechanically with a wax binder. The feedstock had a powder binder ratio of 95:5 (mass%), which was considered as optimum for MIM products of copper. Scanning electron microscopy (SEM) for the feedstock and particle distribution is displayed in Figure 1.

### 2.2. Moldflow Simulation

**Figure 2:**
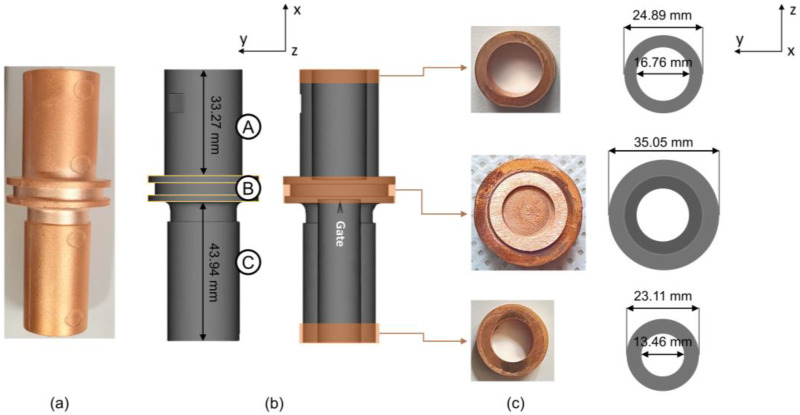
(**a**) Final product, (**b**) Moldflow model of the green part, and (**c**) final product and Moldflow model along *x*-axis. A, B, and C represent big-diameter cylindrical, ring (injection site), and small-diameter cylindrical part, respectively.

A computational analysis was carried out using Moldflow Plastic Insight for the second stage of the MIM process, which produced the green part in the form of the outlet-combo charger terminal body. A three-dimensional model was generated based on the product shown in Figure 2a as the *stp* file format and then imported into software to be analyzed. The injection temperature and pressure were set to ~180 °C and ~5 MPa, respectively. Following this, the gate position was adjusted accordingly. As shown from the actual model in Figure 2b, the green part consists of three subdividing areas, namely big-diameter cylindrical, ring, and small-diameter cylindrical, which hereafter are assigned as the A, B, and C part, correspondingly. Dimensions from the Moldflow model and gate position are provided (Figure 2b) from the view along the *z*-axis. The view along the *x*-axis is provided in Figure 2c. The gate position was designed with the gate simulator feature on Moldflow software (version 45.0.211.0).

### 2.3. Metal Injection Molding (MIM)

The feedstock was injected into the mold cavity using an injection temperature of ~180 °C and an injection pressure of ~5 MPa. The feeding system consisted of one specific gate, as shown in Figure 2b. For the surface analysis, interior analysis, and hardness analysis, the final product was produced. To manufacture the final product, the green part underwent the debinding process at 730 °C for 1 h to generate a binder-free product (brown product), which then was followed by the sintering process at 1050 °C (below copper melting temperature) for 1 h.

### 2.4. Scanning Electron Microscopy (SEM)

The surface observation of the final product was assessed using SEM, and energy dispersive X-ray spectroscopy (EDS) was performed to investigate the element presence. The sample surface was grinded, polished, and etched. An etching solution was prepared by combining 25 mL of distilled water, 25 mL of NH_4_OH, and 5 mL of H_2_O_2_. The prepared samples were immersed for about 5 to 10 s to reveal the microstructure. The observation was performed using SEM Hitachi S-4800 (Hitachi, Tokyo, Japan).

### 2.5. 3D X-ray Computed Tomography

For 3D X-ray tomography (μCT), the X-ray microscope Xradia 620 Versa (Carl Zeiss, Jena, Germany) was used with a spatial resolution of 0.5 µm, voltage of approximately 30~160 kV, and maximum output of 25 W.

### 2.6. Microhardness

Vickers hardness measurement was performed by means of a Vickers 402MVD (Wilson Instrument, Norwood, MA, USA) hardness-testing machine (Wilson Hardness). The polished samples were indented on the YZ-plane (as shown in Figure 2c) under a load of 500 gf, with a holding time of 10 s. A series of each indentation obtained from the polished surface with a gap of ≈5 mm was recorded.

## 3. Results and Discussion

### 3.1. Moldflow Analysis

The absence of a short shot from detection, as shown in Figure 2, can be attributed to the specific injection parameters implemented in this present work, which effectively contributed to the final shape of the green part [26].

**Figure 3:**
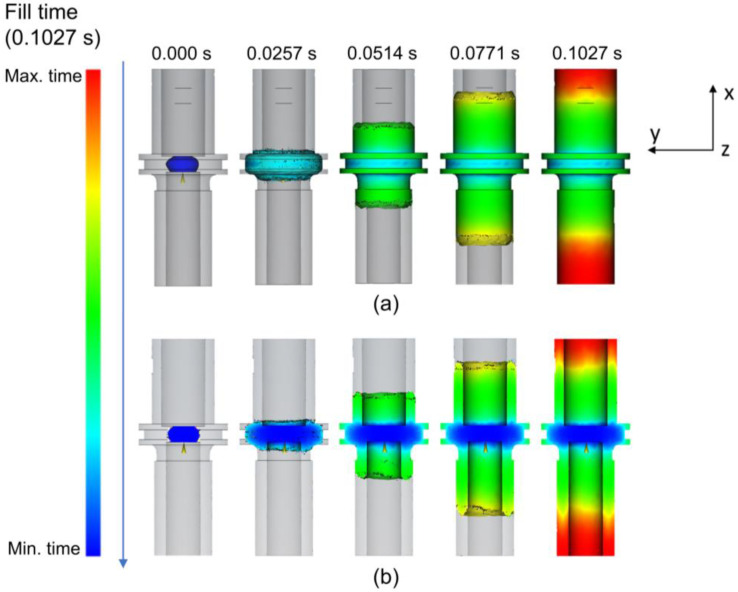
Filling progress with (**a**) the whole body and (**b**) the half-cut body view of the green part.

Figure 3 displays a visual representation of feedstock distribution throughout the progressive filling process for the whole and half-cut body (cutting the XY-plane with a 0.02 mm distance) to explain the outer surfaces and internal region, respectively. This process performed almost simultaneously and was completed in approximately 0.1027 s. Time selection was decided based on the color progression of the mold filling according to the color scale. The color indicates the filling time where blue and red, respectively, mean the minimum and the maximum filling time. In accordance with the gate position, the filling process started from the middle part where the gate was located toward the outer edges of the cylinder in the A and C part. The filling progress showed that the flow directions of the feedstock in the A and C part were opposite each other. Accordingly, the feedstock exhibited the longest filling time at the outermost points of the cylinder during the filling process.

**Figure 4:**
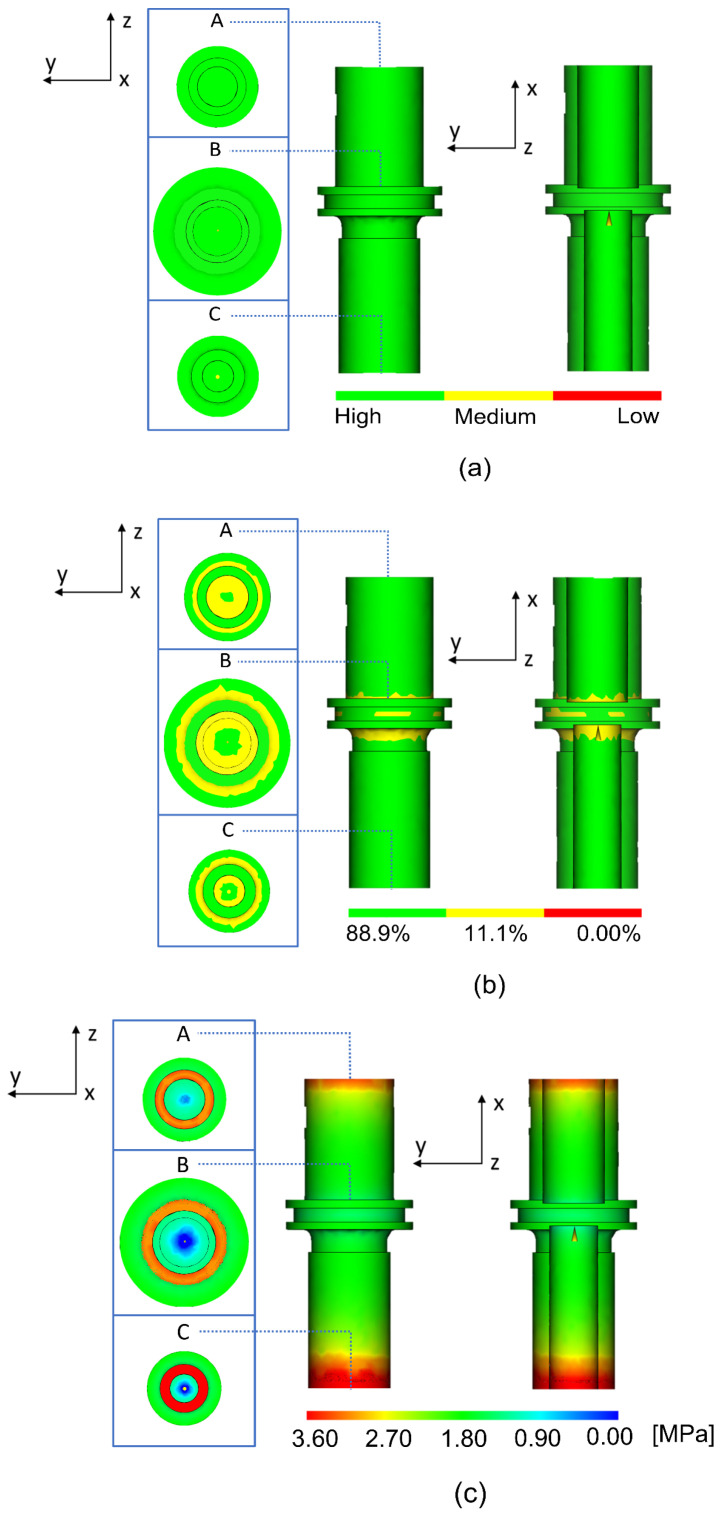
Moldflow modelling results on the (**a**) confidence of fill, (**b**) quality prediction, and (**c**) pressure drop of the A, B, and C part.

Figure 4 presents the confidence of fill, quality prediction, and pressure drop, which were categorized in the fill section of the Moldflow software. Figure 4a shows a high value in terms of confidence of fill (green color) for the whole and half-cut body of the green part. This suggested that the green part will definitely be completely filled and may not have quality problems [27]. Furthermore, simulation on the quality prediction and pressure drop was carried out. The B part shows a noticeable difference in terms of the quality prediction value, which might be attributed to a higher level of complexity as compared to A and C (Figure 4b) [28]. The color code defined the estimated quality for the green part, such that 88.9%, 11.1%, and 0.00% were the mean values for acceptable, less acceptable, and unacceptable part quality, respectively [29]. A green part with a decreased value of quality prediction will have a higher chance to develop phase separation [30]. Figure 4c shows the pressure drop especially at the outer edges (as shown in a red color) of the cylinder in both parts A and C, which is likely due to an insufficient injection pressure (pressure limit was set to 3.6 MPa). Interestingly, it is known that the pressure drop is one of the factors that determine the value of confidence of fill.

**Figure 5:**
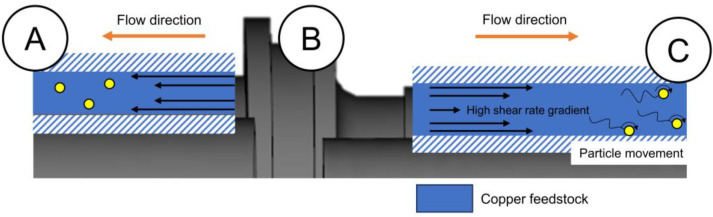
Schematic illustration of the feedstock flow affecting the particle along the cylinder in the A and C part on the green part.

The computational analysis conducted in this study is associated with the specific parameters employed, especially the gate location, injection pressure, and injection temperature. The decision to select the gate position at the B part was two-fold. First, the B part was considered as the most sophisticated shape as compared to the other two parts. It was suggested that positioning the gate near the part with a high sophistication would minimize the possibility of an undesirable phenomenon happening [28,31,32]. Secondly, the gate position at the center of the sample allows the feedstock to flow in two opposite directions. Figure 5 provides the schematic illustration of feedstock flow affecting the particles. It can be seen that the feedstock in the A and C part flew in opposite directions along the cylinder walls due to the gate position, which was expected to result in a decent metallic powder distribution due to its symmetry [33]. Unfortunately, due to the different geometry, where the cylindrical wall in the C part (~9.65 mm) was thicker than the A part (~8.13 mm), the feedstock flow in the C part is likely to exhibit a higher shear rate gradient as compared to that in the A part. The difference in the shear rate gradient between the A and C part will be explained in detail in the following paragraph.

Since the sample design used in this work suffered from the occurrence of the pressure drop during the MIM process, the injection pressure of ~5 MPa was selected despite the fact that the pressure was outside of the typical low pressure range (10 to 500 kPa) used in MIM for metals with a low viscosity [34]. An injection pressure outside the suggested range has been reported to successfully produce a defect-free sample but with adjustment in other parameters (for the current work, the gate and temperature are adjusted) [35]. The consequence of increasing the pressure likely increases the viscosity of the copper feedstock with pseudoplastic behavior [36], as suggested by previous research [37]. A copper feedstock with an increased viscosity would exhibit an area with a shear rate gradient, which then triggers particles to move away from such an area, leading to powder–binder separation [28]. In this study, such a phenomenon would likely occur in the C part due to its larger thickness of the flow channel (as compared to the A part), promoting a high shear rate gradient. A prospect for the occurrence of powder–binder separation would be an additional problem together with a pressure drop, which both existed in the C part.

To alleviate the aforementioned problems, the injection gate was positioned so that it was pointed from the C part toward the B part as shown in Figure 2b. According to the proposed model about the influence of filling patterns on the powder–binder separation in powder injection molding, it was likely that binder separation would occur toward the side the gate pointed to [38,39,40]. Accordingly, the gate position used in this study was placed so that it pointed toward the A part, which would intentionally direct the unavoidable powder–binder separation to the end of the A part. This would balance the powder–binder separation between the A and C part, preventing the C part from suffering powder–binder separation even more, which can potentially lead to crack formation. Such a strategy can be applied to the MIM processing of a pseudoplastic feedstock with a similar design to this study, which had the gate positioned at the center and involved a drop in terms of pressure during the molding process. Lastly, the selection of the molding temperature was led by the principles of the compacting filling pattern theory in powder injection molding. The aim was to achieve optimal feedstock mobility by choosing a temperature that promotes an effective flow and filling during the molding process, as well as homogeneity at the range between 170 °C and 180 °C [38]. The surface and interior investigation was characterized via scanning electron microscopy (SEM) and X-ray computed tomography (µCT), respectively.

### 3.2. Surface Observation

**Figure 6:**
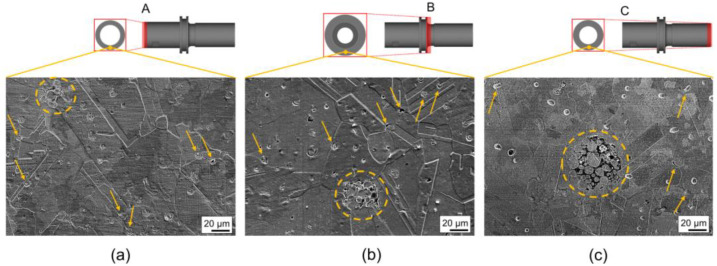
SEM images at the region of interest for the (**a**) A, (**b**) B, and (**c**) C part of the final product.

The surface observation was carried out to know the existence of inhomogeneities such as cracks, large pores, pore agglomeration, and layer delamination (exclusive only for Binder Jetting) [41]. The surface observation on the final product (Figure 6) revealed the existence of a defect in the form of pores (pointed to by arrows), as well as pore agglomeration (marked in the dashed circles).

In general, due to the complex state of stress and abrupt change in viscosity during the extrusion of the feedstock, pores will likely be formed [42]. Pore formation was also reported to be driven by the significant dissipation of thermal energy during the melting process, primarily attributed to the elevated thermal conductivity of copper [43]. In addition, based on its shape, the defect can be categorized as ‘extrusion voids’, which formed on the surface of the product.

The pores were randomly distributed with a slight tendency toward the grain boundaries (GB) and some pores were observed in close proximity to the grain boundaries. Those near the GB may affect the grain growth rate. Fortunately, during sintering, such pores can be eliminated since the GB act as vacancy sinks [44,45]. Regions containing pores due to agglomeration are highlighted in the A, B, and C part. It can be noticed that the region size for the case of the C part is the largest among the other parts (Figure 6). Such areas are commonly identified in commercially available powder characterized by a broad particle size distribution and an enormous presence of particle agglomerates [46]. It is likely that dynamic particle movement due to a shear rate gradient in the C part (Figure 6c) allowed the clustering of large particles, which resulted in severe agglomeration. Therefore, it is suggested that binder separation indeed occurred in the C part, taking form as agglomeration pores with a wide area.

**Figure 7:**
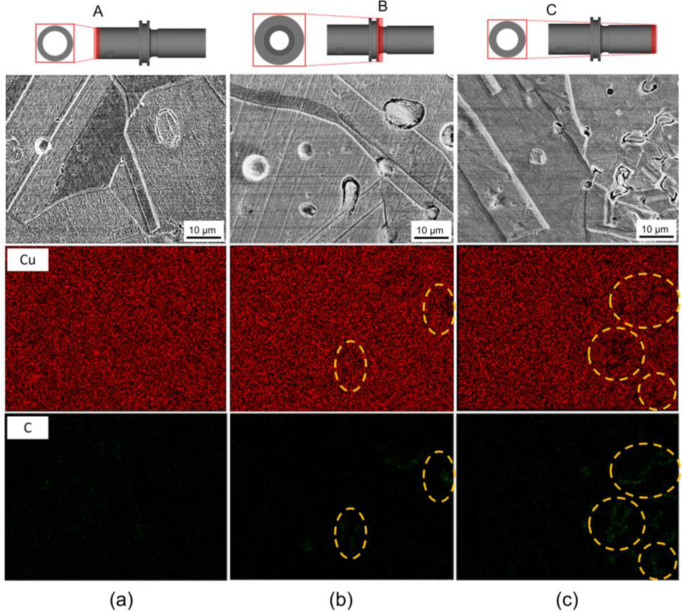
SEM-EDS results at the local region for the (**a**) A, (**b**) B, and (**c**) C part of the final product. The homogenous elemental mapping showing the level of binder separation.

In general, after the debinding stage of the MIM process, residual carbon can be detected due to incomplete binder degradation [26,47]. In this study, it is suggested that the binder aggregate formed by the occurrence of binder separation would be incompletely degraded during debinding, leaving behind a trace of residual carbon. Figure 7 illustrates the SEM-EDS map taken from the A, B, and C part, showing the elemental mapping of copper and carbon elements. Figure 7a demonstrates that the copper element was uniformly distributed in the analyzed region with no observable traces of residual carbon. However, in the B part, several areas (highlighted with the dashed circles) exhibited a low concentration of copper, accompanied by the detection of carbon. This indicates that a certain degree of binder separation occurred in the B part, resulting in the manifestation of residual carbon traces. In the C part, the highest fraction of residual carbon traces was found, indicating the most severe binder separation compared to the A and B part. Fortunately, these regions with binder separation did not result in critical defects such as cracks.

### 3.3. Three-Dimensional Analysis

**Figure 8:**
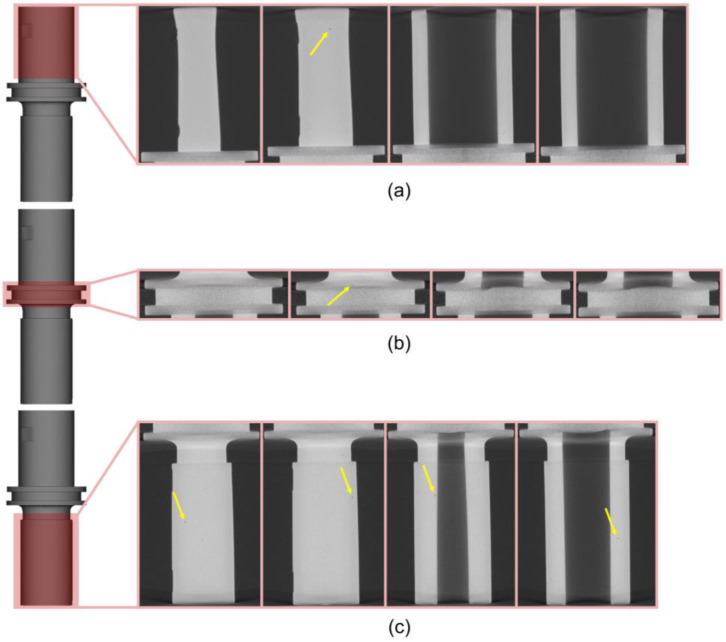
μCT results for the (**a**) A, (**b**) B, and (**c**) C part for the interior of the final product.

Figure 8 shows the interior profiling of the final product via μCT. This method was preferrable since it eliminates the need for destructive operations, such as cutting, mounting, and polishing [48]. From the images, only pores were detected (pointed by arrows) without any trace of cracks in the A, B, and C part. Pores with the size of ~200 μm can be clearly seen, which are mostly found near the surface. Although the definition of an adequate pore size is still debatable, it is reported that those in the range of 100–800 μm are still acceptable [49,50].

During mold filling, especially with a low injection pressure, the fluids near the edges would have an insufficient power to be well integrated with each other. This can potentially result in the formation of cracks at the interface between the solidified feedstock and the feedstock at a higher temperature [51]. In addition, binder separation may trigger the formation of cracks in the interior of the final product [28]. Since no cracks were found, it can be suggested that the molding parameter used in this work successfully alleviated the effect from the pressure drop, which hinders the evolution of binder separation into cracks.

For the case of pore formation occurring in the interior, it was likely due to the trapped air inside the feedstock, which is commonly caused by a vent blockade [51]. Regarding the pores that were observed near the surface, they might be associated with the application of a sufficient pressure. The pressure drives the pores entrapped inside the green part to surpass the surface energy driving force, allowing these pores to diffuse into the surface of the part [52].

### 3.4. Microhardness

**Figure 9:**
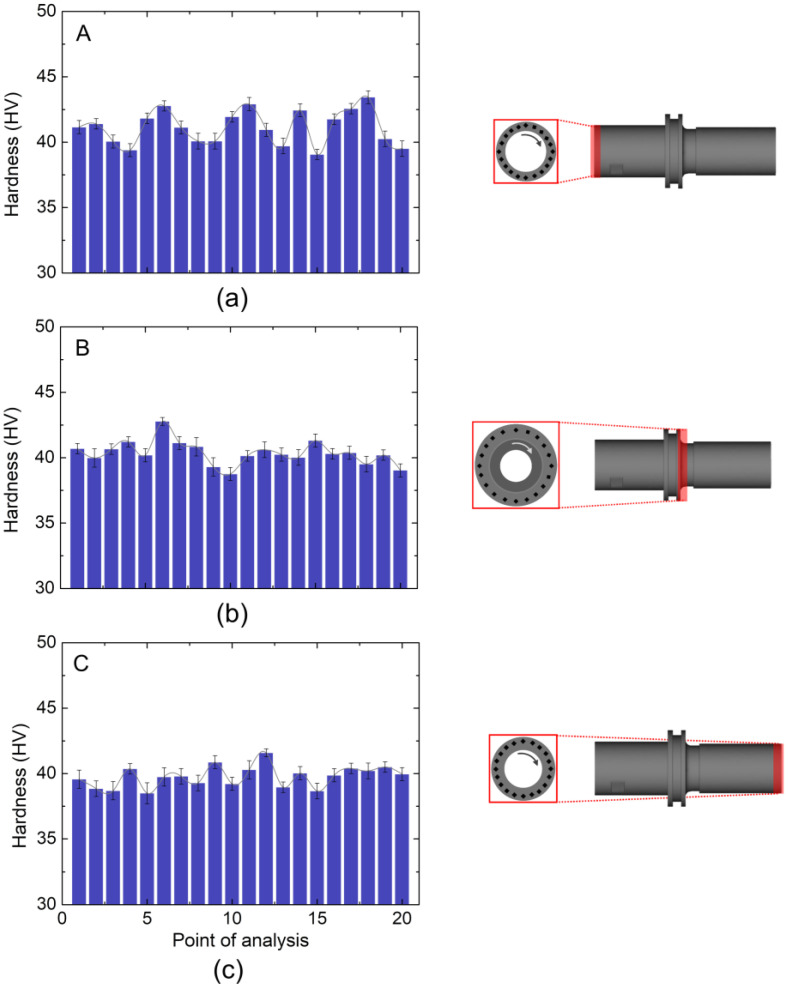
Microhardness distribution on the regions of interest in the final product regarding the (**a**) A, (**b**) B, and (**c**) C part.

It is generally known that the presence of inhomogeneities can have a detrimental impact on the degradation of the mechanical qualities of the finished product [45]. Consistent with the Moldflow analysis findings, a microhardness analysis series was carried out at the regions of interest to generate 20 indentation marks (Figure 9).

As shown in Figure 9a, the A part had the highest average of the hardness value among the other parts, which is 41.12 ± 2.1 HV. For the B part (Figure 9b), it exhibited an average hardness value of 40.36 ± 3.2 HV with a better uniformity than that in the A part. As shown in Figure 9c, the C part had the lowest average hardness value as well as the highest error of 39.76 ± 4.9 HV.

The *p*-value was calculated based on the F value, mean square, sum. square, and degree of freedom (Df) from both the independent variables and the error [53]. A one-way ANOVA (Table 1) revealed that the *p*-value for the hardness in the three different positions was ~0.000501 (much less than 0.05), which means that there was a statistically significant difference among the three different positions.

A post hoc Tukey test (shown in Table 2) was carried out to find out which pair of different positions (A-B, A-C, and B-C) was different, and it was found that only pair A-C showed a significant means difference at the level of 0.05.

The existence of porosity likely affects hardness values and their distribution [31,54,55]. However, the lowest average hardness in the C part as compared to the other parts was likely due to the occurrence of mild binder separation. Although no cracks were formed, it is likely that binder separation induced the formation of pore agglomeration with a relatively wider area. Therefore, it is suggested that jet milling should be utilized to prepare a copper feedstock prior to the MIM process in order to minimize the occurrence of agglomeration. A further investigation into the parameter of debinding and sintering stages would be necessary to further improve the quality of the final product. Specifically, to tackle the problem related to binder separation, an in-depth investigation of the filling pattern during injection molding must be carried out.

In brief, four different fundamentals can be inferred from the current work and might be implemented into other MIM systems. Firstly, the positioning of the gate in a way that ensures homogeneity in the distribution of the feedstock and pressure effects in all directions. Secondly, increasing the injection pressure outside the recommended range to enhance the flow rate and minimize pressure drops on both ends, while controlling the other parameters. Thirdly, finding a suitable temperature was of key importance to ensure the decent filling pattern of the feedstock and suppress early sedimentation for controllable solidification. Lastly, directing the gate appropriately to promote a uniform filling process and mitigate issues related to binder separation.

## 4. Conclusions

The investigation of MIM parameters such as the injection temperature of ~180 °C, injection pressure of ~5 MPa, and single gate was carried out by using Moldflow in the form of the confidence of fill, quality prediction, and pressure drop at three different parts, namely the A, B, and C part. Moldflow results revealed that the area of interest was identified at the edge of the A and C part due to the pressure drop, whilst the B part exhibited a poor quality prediction, which is responsible for the development of defects. The SEM and 3D X-ray showed that the defects were in the form of pores and pore agglomeration, while critical defects such as cracks were not found. Moreover, microhardness characterization revealed that the three parts exhibited a reasonable uniformity, in line with both simulation and microstructural evidence. Thus, the current approach of metal injection molding could be useful to establish guidelines for the MIM of pure copper with a complex shape.

## Figures and Tables

**Table 1:** One-way ANOVA to calculate *p*-value.

	Df	Sum. Square	Mean Square	F Value	*p*-Value
Position	2	18.44	9.22	8.708	0.000501
Error	57	60.35	1.06		

**Table 2:** Post hoc Tukey test to determine the significance of hardness between A, B, and C part.

Pair	Means Difference	Significance (at the Level of 0.05)
A-B	0.76	not significant
A-C	1.35	significant
B-C	0.59	not significant

## Data Availability

The data presented in this study are contained within the article.
